# Unusually warm Indian Ocean sea surface temperatures help to arrest development of El Niño in 2014

**DOI:** 10.1038/s41598-018-20294-4

**Published:** 2018-02-02

**Authors:** Lu Dong, Michael J. McPhaden

**Affiliations:** 1 NOAA/PMEL, Seattle, Washington, USA; 2 Atmospheric Sciences and Global Change Division, PNNL, Richland, Washington, USA

## Abstract

In early 2014, strong westerly wind bursts (WWBs) and high heat content in the equatorial Pacific favored development of a major El Niño. However, significant coupling between the Pacific Ocean and atmosphere failed to take hold during boreal summer of 2014 such that only borderline El Niño conditions were evident by the end of the year. Observational analysis suggests that warm sea surface temperatures (SSTs) in the Indian Ocean in 2014 weakened westerly wind anomalies in the Pacific and may have helped to arrest the development of the El Niño. We test this hypothesis using an ensemble of coupled numerical experiments in which observed Indian Ocean SST anomalies in 2014–15 are prescribed but the Pacific Ocean-atmosphere system is free to evolve. Results confirm that warm SST anomalies in the Indian Ocean created conditions that would have favored a weakening of El Niño by suppressing the Bjerknes feedback in boreal summer of 2014. This process does not preclude others that have been proposed in the unusual evolution of El Niño SSTs in 2014, but it adds to the list a forcing mechanism external to the Pacific basin.

## Introduction

The El Niño–Southern Oscillation (ENSO) is the most prominent year-to-year fluctuation of the earth’s climate system, affecting patterns of weather variability worldwide. Consequently, the scientific community has invested considerable effort over the past 50 years to improve our understanding of and ability to predict ENSO events. Westerly wind bursts (WWBs) over the western Pacific are an episodic wind stress forcing for ENSO, which force eastward currents and downwelling Kelvin waves to initiate an ENSO event^1^. In boreal spring of 2014, strong WWBs over the western Pacific and anomalous high upper ocean heat content in the tropical Pacific Ocean favored development of a major El Niño^2^ and there was widespread speculation among climate scientists and forecasters that a major El Niño was on the way^3,4^. However, subsequent WWBs turned out to be fairly weak^5,6^ and a strong easterly wind surge occurred in boreal summer of 2014^7–9^. While these developments were a likely proximate cause for the weakening of the developing El Niño, other candidate mechanisms that may be related to, or in addition to, these factors have been proposed.

It has been suggested for example that cold SST anomalies in the subtropical Pacific Ocean induced strong northward winds in the tropical Pacific that favored easterlies along the equator^4,10–12^. Another possible factor may have been the effect of a cold background state in the Pacific associated with the negative phase of Pacific Decadal Oscillation (PDO), which is the dominant mode of North Pacific SST variability on decadal timescales^13^, that existed from around 1999 through early 2014^2,14^. The tropical Atlantic Ocean^15^ and Indian Ocean^16–19^ can also affect ENSO via atmospheric teleconnections, though so far it has not been demonstrated that either of these basins had an influence on the Pacific in 2014. For the Indian Ocean in particular, the Indian Ocean Dipole (IOD) and Indian Ocean Basin mode (IOB), characterized by a zonal dipole pattern and basin-wide pattern in anomalous SST, respectively, are the two most prominent modes of interannual variation. Most previous studies have focused on either how the IOD can help initiate ENSO events; or how the IOB, which is a forced by peak ENSO conditions, contributes to the demise of El Niño events by inducing equatorial easterly wind stress anomalies over the tropical Pacific^18,20–24^. Lim and Hendon^25^ have also suggested a negative IOD helped to initiate the weak 2016 La Niña.

In this study, we aim to explore the potential effects of Indian Ocean SSTs on arresting the development of what was anticipated to be a major El Niño in 2014. For most of 2014, the IOB was in a positive (warm) phase and the IOD was in negative phase (unusually warm in the east and cold in the west Indian Ocean)^2^. This situation is atypical of Indian Ocean conditions that have traditionally been viewed as influencing ENSO evolution, i.e. a positive IOD occurring during the development phase of El Niño and positive IOB in the decaying phase^17,26–28^. Considering this particular relationship between Indian and Pacific SSTs in 2014, we hypothesize that the unusually warm Indian Ocean SST was a factor in the failure of a major El Niño to develop in 2014. In the following sections we will motivate this hypothesis more precisely through examination of observations and test it through a series of numerical experiments.

## Results

### Evolution of SST in the Indian and Pacific Ocean in 2014–15

**Figure 1:**
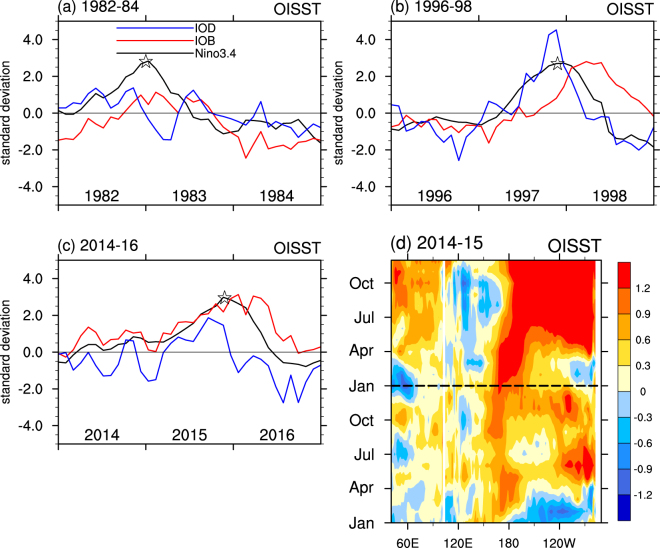
Evolution of SST in the Indian and Pacific Ocean in 2014–15. Time series for monthly Nino3.4 (5°S-5°N, 170–120°W, black lines), IOB (20°S-20°N, 40–120°E, red lines) and IOD (difference between 5°S-5°N, 50–70°E and 10°S-Eq, 90–110°E, blue lines) during (**a**) 1982–1984, (**b**) 1996–1998 and (**c**) 2014–2016 from OISST. All the time series are normalized by their standard deviations. (**d**) Monthly SST anomalies (shaded, in °C) averaged over 5°S-5°N during 2014–2015 based on OISST. The peak of each El Niño is marked by star in (**a**–**c**). The plot was created by NCAR Command Language^45^.

On interannual timescales, ENSO is the dominant mode in the Pacific, while IOB and IOD are the dominant modes in the Indian Ocean. The Nino3.4, IOB and IOD indices (Fig. 1) characterize the evolution of these modes for three extreme El Niño periods observed in 1982–84, 1996–98, and 2014–16. Prior to the peak of the El Niños in late 1982, 1997 and 2015, the IOD was in positive phase, while following the peak of El Niño, the IOB was in positive phase, consistent with the typical relationship between Indian and Pacific SSTs as inferred from previous studies^26–28^. In contrast, in 2014 a warm IOB occurred before boreal winter and lasted for the entire year, while the IOD was negative in boreal summer of 2014. The spatial pattern confirms that the Indian Ocean SSTs show basin-wide warm anomalies with stronger magnitudes in the eastern than western basin from May to September of 2014 (Supplementary Fig. S1). Thus, in 2014 Indian Ocean SSTs did not exhibit conditions typical of those prior to the onset of previous El Niño events. In addition, the SST anomalies in the eastern Pacific stopped warming in boreal summer of 2014 (Fig. 1d). Results from ERSST and HadISST (Supplementary Fig. S2) show consistent results with those from OISST (Fig. 1). The unusual SSTs in the Indian Ocean during 2014 motivate our hypothesis about their role in helping to arrest the development of El Niño conditions in the Pacific that year.

### Atmospheric Anomalies over the Tropical Indo-Pacific in 2014

**Figure 2:**
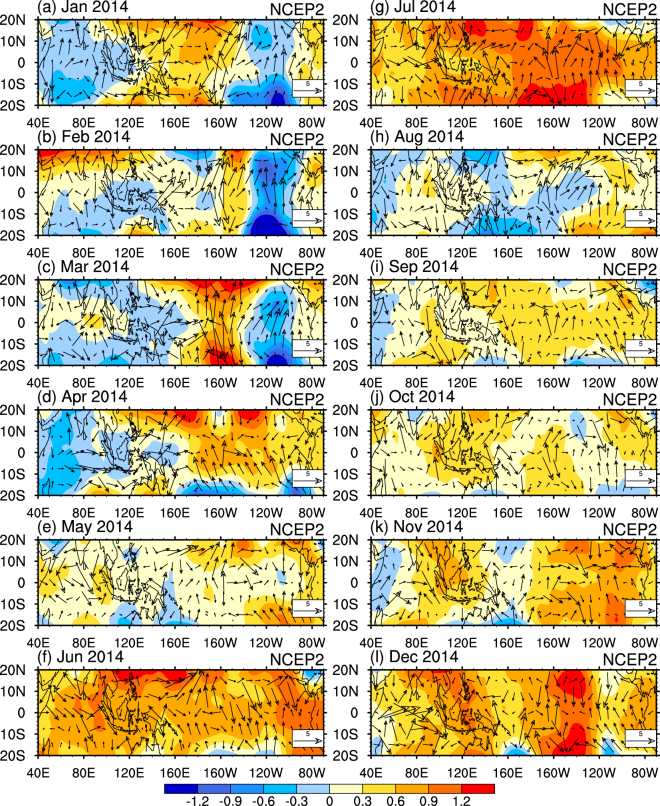
Atmospheric anomalies over the tropical Indo-Pacific in 2014. Monthly anomalies of 1000hPa wind (vector, in m s^−1^) and upper tropospheric temperature averaged over 500–200 hPa (shaded, in °C) for 2014 based on the NCEP2 reanalysis^39^, with the climatological mean for 1982–2011 removed. The plot was created by NCAR Command Language^45^.

In this section, we examine atmospheric circulation anomalies to help determine what distinguished conditions observed in 2014. We use the 1000 hPa wind field to represent low-level circulation, and upper tropospheric temperature averaged over 500–200 hPa to represent the effects of atmospheric heating (as in previous studies^21,29^). Before April 2014, westerly wind anomalies at 1000 hPa cover the equatorial western Pacific, and there is a significant zonal gradient in upper tropospheric temperature over the Indo-Pacific region, which indicates a weakened Walker circulation (Fig. 2a–d). However, from May uniform upper tropospheric temperature in the tropics was indicative of a near-normal Walker circulation (Fig. 2e–g) in which westerly wind anomalies over the western Pacific weakened and shifted toward weak easterly compared to earlier in the year. Focusing on May 2014 when the westerly wind anomalies began to weaken over the western Pacific, we observe positive anomalies in precipitation over most of the Indian Ocean (Supplementary Fig. S3a) coincident with the transition to warm upper tropospheric temperature (Fig. 2e). These tendencies are consistent with the idea that enhanced convection due to SST warming in the Indian Ocean induced local low-level atmosphere convergence and contributed to the weakened westerly wind anomalies over the western Pacific.

For comparison, in 2015 the anomalous east–west SST contrast (Fig. 1d) and related zonal gradient in upper tropospheric temperature over the Indo-Pacific region (Supplementary Fig. S4) existed all year-round, which reinforced westerly wind anomalies over the western Pacific (Supplementary Fig. S4). The sustained westerly wind anomalies fed back to the SST zonal gradient to cause further weakening of the trade winds, a process that is referred to as the Bjerknes feedback^30^. This positive feedback amplifies initial warm SST anomalies in the equatorial eastern Pacific in such a way as to generate El Niño events^1,31^. Our hypothesis is that the unusually warm Indian Ocean SSTs favored weakened westerly wind anomalies in the western Pacific in boreal summer of 2014, which contributed to the failure of the positive feedbacks between surface winds and SSTs to develop in the Pacific^2^. Thus, growth of the developing El Niño was arrested such that only borderline warm ENSO conditions existed by the end of 2014.

### The Effects of Indian Ocean SSTs on Developing El Niño Conditions during 2014–15 Based on Numerical Experiments

**Figure 3:**
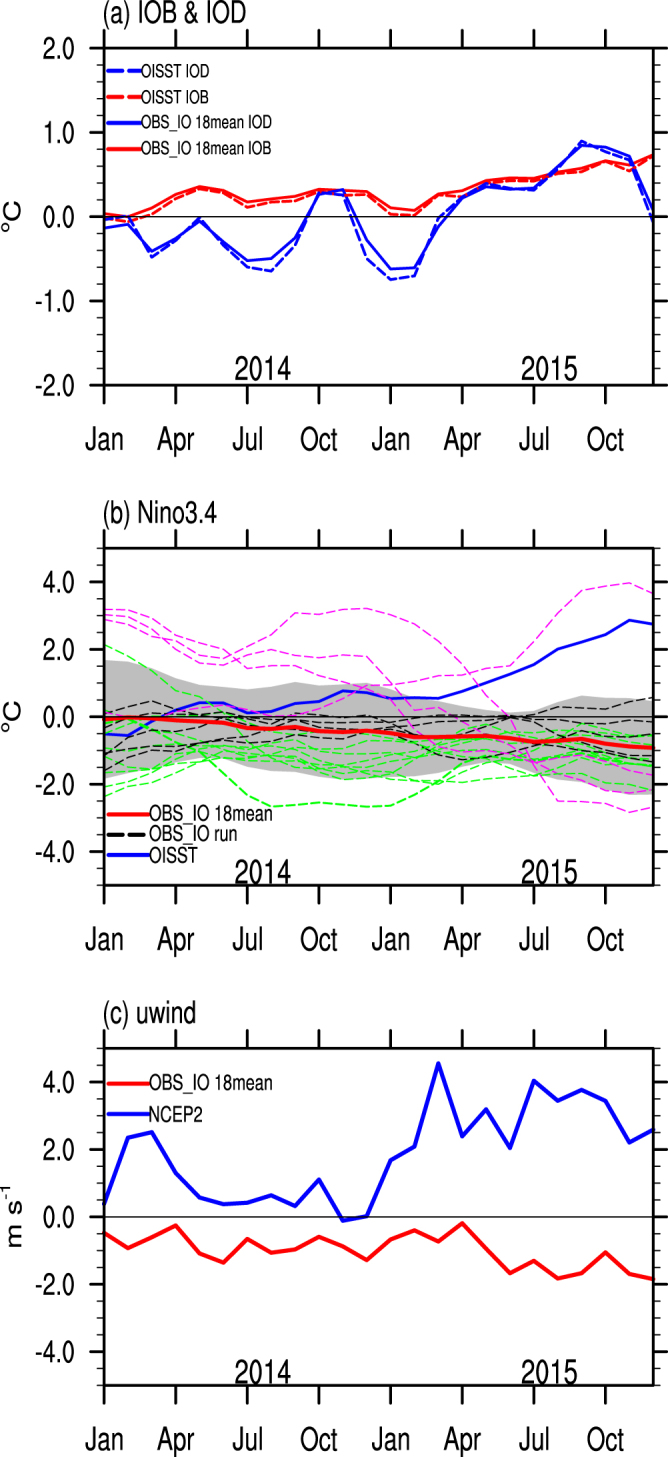
The effects of Indian Ocean SSTs on Nino3.4 and zonal wind in the Pacific based on *OBS_IO* runs. (**a**) Time series of monthly IOB (red lines, in °C) and IOD (blue lines, in °C) during 2014–2015 based on OISST (dashed) and the ensemble mean of 18 *OBS_IO* runs (solid). (**b**) Time series of monthly Nino3.4 (in °C) during 2014–2015 based on OISST (blue line), each member of *OBS_IO* run (dashed lines) and the ensemble mean of 18 *OBS_IO* runs (red line). The dashed lines identify runs that exhibited either El Niño (magenta) and La Niña (green) conditions during the period November 2014-February 2015 (See Methods Section for definitions of El Niño and La Niña). These runs are chosen for further analysis in the composite Supplementary Figs S3, S7 and S8. The grey shading indicates one standard deviation of the 18 member *OBS_IO* ensemble. (**c**) Time series of monthly zonal surface (1000 hPa) wind anomaly (in m s^−1^) averaged over 150–180°E, 5°S-5°N based on NCEP2 (blue line), the ensemble mean of 18 *OBS_IO* runs (red line). The x-axis denotes the months of 2014–15. All the anomalies are relative to the climatology from the *Control* run. The plot was created by NCAR Command Language^45^.

In this section, two sets of coupled numerical experiments are used to examine the effects of Indian Ocean SST on the developing El Niño. One is a *Control* run, a free coupled simulation for 28 years with external forcing fixed at year 2000. The other is an ensemble of 18 *OBS_IO* runs, in which Indian Ocean SST anomalies are prescribed as observed in 2014–15 but starting from different initial conditions based on the *Control* run. Note that the ensemble mean of 2-year *Control* runs, whose initial conditions are the same as those of the 18 *OBS_IO* runs, exhibits negligible Nino3.4 SST anomalies compared to climatology (Supplementary Fig. S5). This result indicates that the ensemble mean of 18 *OBS_IO* runs succeeds in suppressing the internal variability to highlight Indian Ocean impacts. We first evaluate the performance of *OBS_IO* run by comparing the IOB and IOD with observations (Fig. 3a). The consistent evolution of the IOB and IOD between the observation and *OBS_IO* indicates that we succeeded in constraining Indian Ocean SST anomalies in the model to the observations, in particular to a positive phase of the IOB throughout 2014–15 (red lines) and a negative phase of the IOD in the boreal summer of 2014 (blue lines). Time series of Nino3.4 index in *OBS_IO* runs show large spread in Nino3.4 index for different *IOB_IO* members (Fig. 3b), suggesting that the warm Indian Ocean SST anomalies in 2014 did not guarantee the suppression of El Niño. Strong stochastic WWBs and other factors can overwhelm the Indian Ocean’s effect and still allow for the development of an El Niño event, as happened in 2015^6^. However, the observed Nino3.4 SST anomaly in 2014 is within one standard deviation of the 18 *OBS_IO* runs, indicating the important role of Indian Ocean SSTs in helping to arrest the El Niño in 2014 (Fig. 3b). The ensemble mean of all *IOB_IO* runs also identifies the tendency of warm Indian Ocean SSTs to favor La Niña-like SST anomalies in the Pacific from April 2014 until the end of 2015 (red line in Fig. 3b).

To explore the effects of Indian Ocean SST on Pacific conditions as occurred 2014, we choose three *OBS_IO* runs that have similar Nino3.4 index values to those observed in January 2014 (Supplementary Fig. S6). All the three simulations show negative anomalies in Nino3.4 index from July of 2014, when Nino3.4 stopped increasing in the observations. This result further supports our conclusion that observed Indian Ocean SST anomalies contributed to the weakening El Niño in boreal summer of 2014.

The monthly zonal surface wind averaged over 5°S-5°N, 150–180°E can be regarded as an indicator for the strength of the wind anomalies in the western Pacific^16^. The westerly anomalies were fairly weak in boreal summer of 2014 (red line in Fig. 3c). At the same time, Indian Ocean SSTs favored easterly wind anomalies during 2014–15 (blue line), opposing the tendency towards westerly wind anomalies in the Pacific. Indian Ocean SSTs are just one factor affecting the evolution of ENSO, so the warm Indian Ocean SST anomalies did not completely kill the developing El Niño however. Indeed, the observed Nino3.4 SST anomaly in 2015 is greater than one standard deviation of the *OBS_IO* runs (Fig. 3b), indicating that the role of Indian Ocean SSTs in 2015 was overwhelmed by other factors. Warm Pacific SSTs, high ocean heat content, and a new spate of WWBs in early 2015 were enough to intiate a full blown El Niño by mid-2015^6,8^.

**Figure 4:**
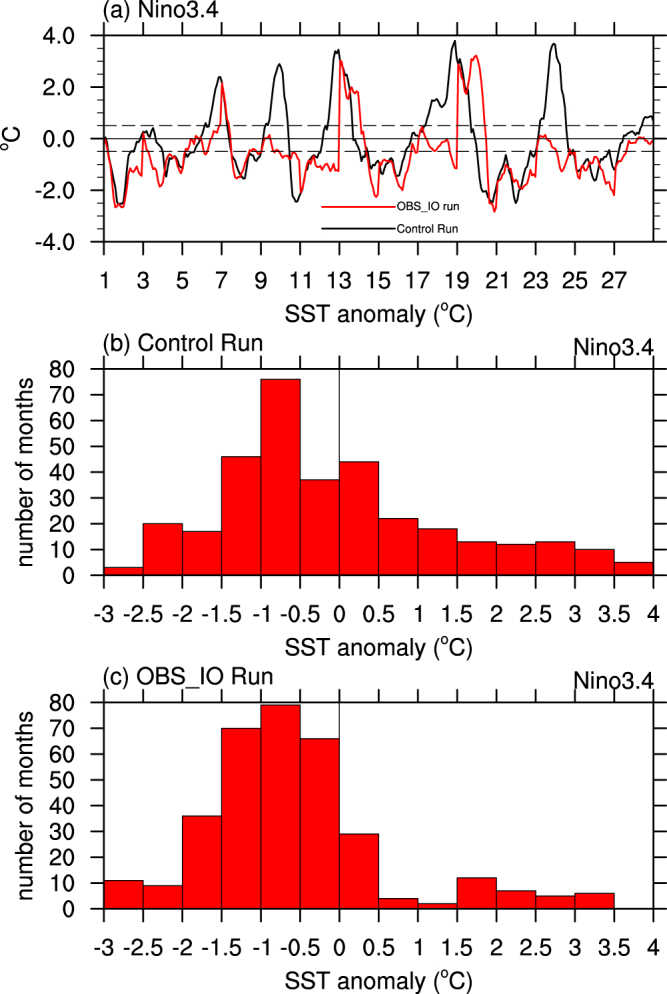
The likelihood of producing El Niño events between *Control* run and *OBS_IO* run. (**a**) Time series of monthly Nino3.4 index (in °C) for the 28-year *Control* run (black line) and the *OBS_IO* run (red line) initiated on the first day of year 1, 3, 5, 7, 9, 11, 13, 15, 17, 19, 21, 23, 25 and 27 of *Control* run. The x-axis denotes the year number. Histograms of monthly Nino3.4 anomalies (in °C) for the 28-year (**b**) *Control* run and (**c**) *OBS_IO* run. The plot was created by NCAR Command Language^45^.

Time series and probability distributions of the monthly Nino3.4 index are compared between the *Control* run and the *OBS_IO* run (Fig. 4). The distribution of the Nino3.4 index in the *Control* run shows a positive skew, with a small number of extreme positive anomalies and a greater number of smaller negative anomalies in the 28 years (Fig. 4b). These characteristics are consistent with the asymmetric character of SST anomalies in the eastern and central equatorial Pacific, which exhibit positive skewness with stronger El Niños than La Niñas and longer duration of La Niñas than El Niños^18,32^. With observed SST anomalies prescribed in the Indian Ocean, the Nino3.4 index in the *OBS_IO* run shifts towards negative values compared to the *Control* run, with decreased likelihood of El Niño and increased likelihood of La Niña (Fig. 4c). Based on the Kolmogorov-Smirnov two-sample test^33,34^, which can be used to examine whether two samples are drawn from the same distribution, the probability distributions of the Nino3.4 indexes between the *Control* run and *OBS_IO* runs are significantly different at the 99% level of confidence. Note that just having warm Indian Ocean SST anomalies in 2014–15 is not enough to completely suppress the occurrence of all El Niños, as there are still El Niño events (positive anomalies in Nino3.4) in the *OBS_IO* run (Fig. 4c). This diversity of outcomes is consistent with the different behavior observed in 2014 and 2015, both years of which featured warm Indian Ocean SST anomalies. These results indicate that warm Indian Ocean SSTs during 2014–15 would favor suppression El Niño, and likely did so in 2014. A warm Indian Ocean however is not a sufficient condition to suppress El Niño development since other factors, like energetic WWBs that arose in early 2015, can overwhelm the damping effect of unusually warm Indian Ocean SSTs.

**Figure 5:**
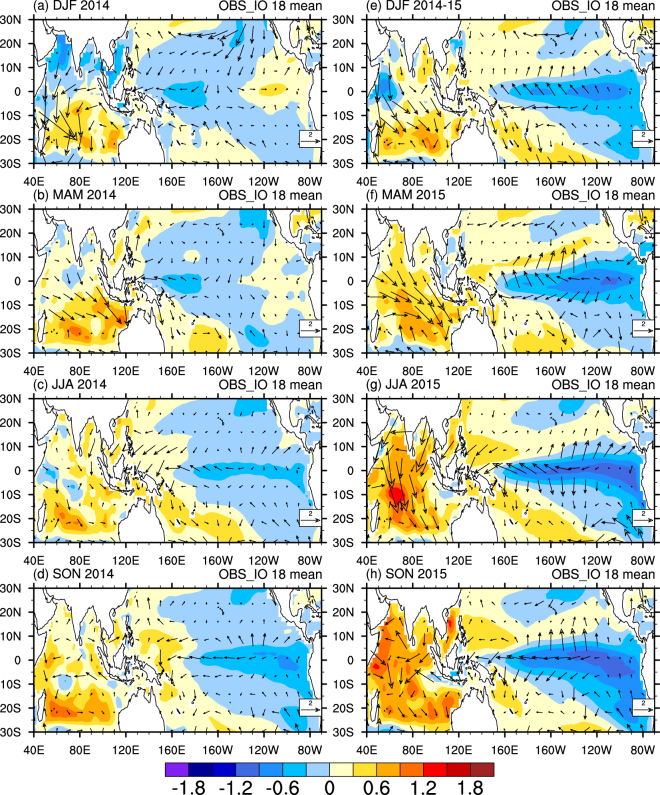
The mechanism of the Indian Ocean SSTs on the development of El Niño. Seasonal anomalies of SST (shaded, in °C) and surface wind at 1000 hPa (vector, in m s^−1^) during 2014–15 based on the ensemble mean of the 18 *OBS_IO* runs. The plot was created by NCAR Command Language^45^.

The spatial pattern of seasonal SST and surface wind anomalies induced by the observed Indian Ocean SST anomalies are presented based on the ensemble mean of *OBS_IO* runs (Fig. 5). Indian Ocean SSTs favored easterly wind anomalies over the equatorial western Pacific in 2014–15. For the transitional month of May 2014, the model developed anomalous precipitation over much of the Indian Ocean in response to the warm SSTs (Supplementary Fig. S3b), consistent with the observations (Supplementary Fig. S3a). This enhanced convection drove anomalous low-level convergence over the Indian Ocean, which is linked to easterly wind anomalies in the western Pacific. Results from the ensemble mean of 9 *OBS_IO* runs with La Niña events in 2014 show even stronger signals (Supplementary Fig. S3c), and further confirm the effects of Indian Ocean. In the absence of any countervailing tendencies, Indian Ocean SSTs induce even stronger La Niña-like SST anomalies in 2015 in the model simulations (Fig. 5e–h). However, outcomes different from the ensemble mean are possible, with some ensemble members developing into El Niño in either 2014 or 2015 (Fig. 3b).

Regarding the large spread of 18 *OBS_IO* runs, we conducted further composite analysis depending on whether El Niño or La Niña developed in the boreal winter of 2014, as indicated in Fig. 3b. The ensemble mean of the 9 La Niña events (Supplementary Fig. S7) shows consistently stronger signals compared to the ensemble mean of the all 18 *OBS_IO* runs (Fig. 5), with enhanced cold anomalies in the eastern Pacific from January to December. For comparison, there are only 4 *OBS_IO* runs that develop into an El Niño event in the boreal winter of 2014 with the unusually warm Indian Ocean SST prescribed (Fig. 3b). In addition, the Pacific Ocean shows El Niño anomalies in the initial conditions in three *OBS_IO* runs (magenta lines in Fig. 3b). The warm anomalies in the Indian Ocean are weaker than those in the eastern Pacific, and the effects of the Indian Ocean are overwhelmed in these three runs (Supplementary Fig. S8), as was observed 2015 (Fig. 1d). Thus, the ensemble mean pattern in Fig. 5 is an indicator of tendencies, not an absolute determinant of outcomes, because of the nature of stochastic forcing in the ENSO cycle. As a result, whether warm Indian Ocean SST anomalies can quash a developing El Niño depends partly on chance as to whether strong WWBs develop. Such observed WWBs did not develop in boreal summer of 2014 whereas they did at the same time of year in 2015^6^.

## Summary and Discussion

The main motivation of the present study is to explore whether pre-existing warm Indian Ocean SST anomalies could have helped to stall the development of the 2014 El Niño. Most previous studies have considered the role of the Indian Ocean in either initiating El Niño events^16,19^ or contributing to their demise once fully developed^18,20–24^. Here we address a different question of how conditions in the Indian Ocean might have suppressed the onset of an El Niño for the unusual case of what turned out to be a borderline event in 2014. Our main conclusions are that: (1) evidence from both observational analysis and numerical experiments indicates that the warm SST anomalies in the Indian Ocean during 2014–15 favored easterly wind anomalies over the western tropical Pacific via enhanced convection and low-level convergence, and induced La Niña-like SSTs across the basin; (2) these warm Indian Ocean SSTs weakened the Bjerknes feedback in the Pacific in boreal summer of 2014 so that only borderline El Niño conditions were evident in boreal winter 2014; (3) SSTs in Indian Ocean are a contributing factor but not necessarily a determining factor in the development of El Niño, since other processes such as strong stochastic WWBs can overwhelm the Indian Ocean’s influence in triggering and amplifying El Niño as occurred in 2015.

We have found that warm Indian Ocean SST anomalies from May to July 2014 played a key role in damping what might have otherwise been a strong El Niño in 2014. One might have anticipated such a result based on previous studies on how Indian Ocean SSTs facilitate the demise of mature El Niño events. To our knowledge though, this study is the first to address the effects of Indian Ocean SSTs on arresting the growth of the incipient 2014 El Niño. Also, Indian Ocean SST anomalies in mid-2014 were not forced from the Pacific, as often occurs during the decaying phase of El Niño. Why Indian Ocean SSTs were unusually warm in early 2014 is thus an interesting question but one that is beyond the scope of this study.

Our conclusion that the Indian Ocean played a role in helping to arrest the development of the 2014 El Niño does not negate other studies assessing competing processes that may have also been at work as mentioned in the Introduction. The various damping mechanisms proposed to date are not mutually exclusive and some or all may have been in play. Our point is that a complete understanding of the unusual circumstances surrounding development of the borderline 2014 El Niño should take into account influences from the Indian Ocean. Moreover, given this remote influence, seasonal forecast models should take into account variability in the Indian Ocean region (among other regions external to the tropical Pacific that can influence ENSO evolution) to improve the predictability of El Niño and La Niña.

## Datasets and Methods

Monthly SST data are obtained from: (1) NOAA Optimum Interpolation SST version 2 (OISST: 1° latitude × 1° longitude)^35^; (2) National Oceanic and Atmospheric Administration Extended Reconstructed SST version 4 (ERSST: 2° latitude × 2° longitude)^36,37^; (3) Hadley Centre Global Sea Ice and Sea Surface Temperature (HadISST: 1° latitude × 1° longitude)^38^. Anomalies in observed atmospheric circulation are estimated using monthly atmospheric reanalysis products from the National Centers for Environmental Prediction–Department of Energy (NCEP2: 1.9° latitude × 1.875° longitude)^39^. All the anomalies in this study are relative to the climatological annual cycle over 1982–2011. The choice of the reference period does not affect our main findings.

To examine the effects of Indian Ocean SST on the developing El Niño, we performed two sets of experiments using the NCAR CESM1.2^40^, which is a fully coupled global state-of-the-art climate system model. Generally speaking, the main characteristics of ENSO cycle and WWBs can be successfully reproduced by CESM model, owning to its improvement in the representation of the deep convection^41,42^. We first conducted a free coupled simulation for 28 years with external forcing fixed at year 2000, called *Control* run. To achieve a better understanding of the effect of Indian Ocean SSTs, we conducted another set of experiments called *OBS_IO*. In these experiments, we prescribed observed daily SST anomalies plus climatological SST from the *Control* run in the Indian Ocean (20°S-20°N, 40–120°E) for 2014–15 with external forcing fixed at year 2000, while other oceans, including the Pacific, were freely coupled to the atmosphere. This approach of prescribing the SST anomalies in a target region to study the effect of those SSTs on other phenomena is a widely used strategy in climate modeling^43,44^ and allows us to determine the reponse of the Pacific Ocean to Indian Ocean SST anomalies. The Indian Ocean SST anomaly was blended with and relaxed to the modeled anomaly in a buffer zone around the Indian Ocean within five grid boxes. We obtained the daily SST anomalies from monthly dataset via linear interpolation by positioning the monthly mean at the middle day of each month, which is commonly done in CMIP5 experiments (http://www-pcmdi.llnl.gov/projects/amip/AMIP2EXPDSN/BCS/). We ran 18 ensemble members for the *OBS_IO* configuration. Each ensemble member was prescribed with the same Indian Ocean SST anomalies from OISST (Supplementary Fig. S1), but began from different initial conditions based on the *Control* run. The first 10 of these *OBS_IO* simulations were initialized from the first time step on January 1^st^ for each two years of the first 20 years of the *Control* run, while the last eight simulations were initialized from the first time step on January 1^st^ for each year of the last eight years of the *Control* run to add more ensemble members and better suppress the internal variations in the *OBS_IO* ensemble mean. All the members were integrated for two years with observed Indian Ocean SST anomalies prescribed as 2014–15. Note that there is an initialization shock in each *OBS_IO* member at the first time step of January 1^st^, which is small and ignored in this study when we focus on the monthly anomalies of the ensemble mean.

Monthly outputs were analyzed with all the anomalies in the *OBS_IO* runs computed relative to the climatology from the *Control* run. Some of the *OBS_IO* runs developed El Niño or La Niña conditions during November 2014 to February 2015. For our purpose, we define El Niño in terms of SST averaged in the Nino3.4 region (5°S-5°N, 170–120°W) exceeding 0.5 °C, and La Niña for Niño3.4 less than −0.5 °C, for at least 3 of the 4 months in November 2014-February 2015, which is when the expected the peak of the El Niño was supposed to occur. We quantitatively test our hypothesis by comparing the likelihood of producing El Niño events between the *Control* run and *OBS_IO* runs. The *OBS_IO* experiments in addition indicate how Indian Ocean SST anomalies are able to alter surface winds and SSTs in the Pacific and thus affect El Niño evolution.

## Electronic supplementary material

Supplementary Information
