# Supplementary material for: Unusually warm Indian Ocean sea surface temperatures help to arrest development of El Niño in 2014
Source: Sci Rep. 2018 Feb 2;8:2249. doi: 10.1038/s41598-018-20294-4 (PMC5797157; doi:10.1038/s41598-018-20294-4)
Supplement: Supplementary file 1 — Supplementary Information [file 41598_2018_20294_MOESM1_ESM.doc]

Unusually warm Indian Ocean sea surface temperatures help to arrest development of El Niño in 2014

Lu Dong^1,2*^, Michael J. McPhaden^1^

*1 NOAA/PMEL, Seattle, Washington, USA*

*2. Atmospheric Sciences and Global Change Division, PNNL, Richland, Washington, USA*

Submitted to ***Scientific Reports***

**(Supplementary Information)**

**Fig. S1.** SST anomalies (in ^o^C) for every month in 2014-15 based on OISST relative to the climatological mean for 1982-2011. We prescribed these anomalies in the *OBS_IO* runs based on CESM1.2. The plot was created by NCAR Command Language^1^.

**Fig. S2.** Time series for monthly Nino3.4 (5^o^S-5^o^N, 170-120^o^W, black lines), IOB (20^o^S-20^o^N, 40-120^o^E, red lines) and IOD (difference between 5^o^S-5^o^N, 50-70^o^E and 10^o^S-Eq, 90-110^o^E, blue lines) during 1981-1983, 1996-1998 and 2014-2016 from (**a-c**) ERSST and (**d-f**) HadISST. All the time series are normalized by their standard deviations. Monthly SST anomalies (shaded, in ^o^C) averaged over 5^o^S-5^o^N during 2014-2015 based on (**g**) ERSST and (**h**) HadISST. The plot was created by NCAR Command Language^1^.

**Fig. S3.** Anomalies of precipitation (in mm day^-1^) and 1000hPa wind (vector, in m s^-1^) for May 2014 based on (**a**) CPC Merged Analysis of Precipitation (CMAP)^2^ and NCEP2, (**b**) the ensemble mean of the 18 *OBS_IO* run members and (**c**) the ensemble mean of 9 *OBS_IO* runs with the winter of 2014 showing La Nina events, shown as green dashed lines in Fig. 3b. The plot was created by NCAR Command Language^1^.

**Fig. S4.** Anomalies of 1000hPa wind (vector, in m s^-1^) and temperature of upper troposphere averaged over 500-200hPa (shaded, in ^o^C) for every month in 2015 based on the NCEP2 reanalysis, with the climatological mean for 1982-2011 removed. The plot was created by NCAR Command Language^1^.

**Fig. S5.** Monthly SST time series for the IOB (red, in ^o^C), IOD (blue, in ^o^C) and Nino3.4 (black, in ^o^C) from the ensemble mean of 18 2-year *Control* runs with the same initial year of 18 *OBS_IO* runs. The shading indicates one standard deviation of the 18 *OBS_IO* member ensemble for each index. The x-axis denotes the months of the two-year *Control* runs. The plot was created by NCAR Command Language^1^.

**Fig. S6.** Time series of monthly mean Nino3.4 SST (in ^o^C) during 2014-2015 based on OISST (black line) and three *OBS_IO* runs (red lines) with Nino3.4 anomalies similar to those observed in January of 2014. The plot was created by NCAR Command Language^1^.

**Fig. S7.** Monthly anomalies of SST (shaded, in ^o^C) and surface wind at 1000hPa (vector, in m s^-1^) for 2014 based on the ensemble mean of the 9 *OBS_IO* runs with the boreal winter of 2014 showing La Niña events, shown as green dashed lines in Fig. 3b. The plot was created by NCAR Command Language^1^.

**Fig. S8.** Same as Fig. S7, but for the ensemble mean of the 4 *OBS_IO* runs with the boreal winter of 2014 showing El Niño events, shown as magenta dashed lines in Fig. 3b. The plot was created by NCAR Command Language^1^.

**References for Supporting Information**

1. The NCAR Command Language (Version 6.4.0) [Software]. Boulder, Colorado: UCAR/NCAR/CISL/TDD. http://dx.doi.org/10.5065/D6WD3XH5 (2017).
2. Xie, P. & Arkin, P. A. Global precipitation: A 17-year monthly analysis based on gauge observations, satellite estimates, and numerical model outputs. *Bull. Amer. Meteor. Soc.* **78,** 2539 – 2558 (1997).
